# Targeted Screening and Quantification of Characteristic Sesquiterpene Lactones in *Ambrosia artemisiifolia* L. at Different Growth Stages

**DOI:** 10.3390/plants13152053

**Published:** 2024-07-25

**Authors:** Balázs Kovács, Péter Püski, Ákos Bajtel, Elek Ferencz, Boglárka Csupor-Löffler, Dezső Csupor, Tivadar Kiss

**Affiliations:** 1 Institute of Pharmacognosy, Faculty of Pharmacy, University of Szeged, 6720 Szeged, Hungary; kovex92@gmail.com (B.K.); peterpuski@gmail.com (P.P.); bajtel.akos@szte.hu (Á.B.); csupor.dezso@szte.hu (D.C.); 2 Department of Physical Chemistry, Faculty of Pharmacy, George Emil Palade University of Medicine, Pharmacy, Science, and Technology of Targu Mures, 540142 Târgu Mureș, Romania; elekferencz@yahoo.com; 3 Institute of Clinical Pharmacy, Faculty of Pharmacy, University of Szeged, 6725 Szeged, Hungary; csuporboglar@gmail.com; 4 Institute of Translational Medicine, Medical School of Pécs, University of Pécs, 7624 Pécs, Hungary; 5 HUN-REN-SZTE Biologically Active Natural Products Research Group, 6720 Szeged, Hungary

**Keywords:** *Ambrosia artemisiifolia*, sesquiterpene lactones, HPLC/DAD, chemical profile

## Abstract

Sesquiterpene lactones are specialized plant metabolites with promising pharmacological activities. These metabolites are characteristic marker compounds for the aerial parts of *Ambrosia artemisiifolia*. Numerus sesquiterpene lactones have been isolated from ragweed; however, there is no information on their bioproduction and quantification throughout the life cycle of the plant. The sesquiterpene lactone content of ragweed samples collected in Szeged and Nyíri was analyzed using HPLC. Significant differences in the amount and bioproduction rhythm of sesquiterpene lactones were found between the two sets of samples. The samples collected near Szeged contained significantly lower amounts of the investigated compounds compared to the Nyíri samples. Sesquiterpene lactone production in the samples peaked at the end of July or in August; the trend of the change in sesquiterpene lactones might correlate with precipitation and temperature. Geographical location and geoclimatic factors might exert significant influence on the production of sesquiterpene lactones in ragweed.

## 1. Introduction

*Ambrosia artemisiifolia* L., commonly known as ragweed, annual ragweed, and low ragweed, belongs to the Asteraceae family. It is distributed in the southwestern United States and nearby north Mexico with a center of origin in the Sonoran Desert [1]. Ragweed is the most successful and widespread species of the *Ambrosia* genus and has been reported throughout the European continent (Hungary, France, Switzerland, Germany, Russia, and the former Yugoslavian republics) [2]. As a pioneer weed, its wide ecological niche explains its fast spreading in mostly ruderal or disturbed habitats, as do the geoclimatic conditions of the aforementioned territories. Some authors have hypothesized that global warming and the genetic variability of ragweed could accelerate the infection of new territories [3,4,5].

As an annual weed, it produces a large amount of highly allergenic pollen, which can cause hay fever [6]. The pollen contains two main allergens, Amb 1 and Amb 2 endopeptidase with immunoglobulin-E binding capacity, and they can trigger rhinitis, oculorhinitis, or other symptoms of hay fever in humans [7,8]. Dermal exposure to the plant can cause contact dermatitis, which has previously been described for other species of Asteraceae. The reaction is caused by sesquiterpene lactones, which are characteristic specialized metabolites of this family [9].

*A. artemisiifolia* sesquiterpenes are structurally diverse secondary metabolites with a 15-carbon-atom skeleton. To date, 60 sesquiterpene lactones have been isolated from *A. artemisiifolia,* and most reports have described the guaiane, pseudoguaiane, seco-pseudoguaiane, germacrane, daucane, and eudesmane sesquiterpene backbones in samples of different geographical origins. In addition to primary bioactivities (cytotoxic and antiproliferative activity), these specialized metabolites also have other notable pharmacological activities, such as antibacterial, antifungal, anti-inflammatory, and antiprotozoal effects [10]. The sesquiterpene lactone content can vary over time and in the different vegetation periods of the plant [11].

The medicinal use of the herb has started recently in Europe, even though common ragweed had never been part of folk medicine, and to the best of our knowledge, the long-term consumption of the plant has never been examined. In an animal experiment, the repeated use of ragweed was nephrotoxic and had controversial effect on brain tissue. It was hypothesized that these effects are related to the presence of cytotoxic sesquiterpene lactones in *A. artemisifiifolia.* Based on these alarming results and due to the lack of epidemiological or clinical evidence of safety, the use of this herb as a medicinal plant is questionable [12]. To assess the potential benefits or risks of the herb, the identification and quantification of the main sesquiterpene lactone content in the aerial parts of the plant from different geoclimatic origins and in different vegetation periods is needed. For such purposes, we developed an HPLC-DAD method for the analysis of *A. artemisiifolia* sesquiterpene lactones, and two sets of samples with different geographical origins were analyzed.

## 2. Results

### 2.1. HPLC/DAD Method for the Analysis of A. artemisiifolia Aerial Parts

An HPLC/DAD analysis was carried to qualitatively and quantitatively analyze five marker compounds, i.e., psilostachyin (**1**), peruvin (**2**), acetoxydihydrodamsin (**3**), costunolide (**4**), and isoalantolactone (**5**) (Figure S1), from the aerial parts of *A. artemisiifolia*, collected in two different regions of Hungary from June to October. The developed HPLC method allowed for the reliable analysis of these compounds in the ragweed samples (Figure S2). The analyzed sesquiterpene lactones were identified based on retention time and UV absorbance. The elution order of the observed compounds was as follows: psilostachyin (**1**, R_t_ = 7.17 min), peruvin (**2**, R_t_ = 8.25 min), acetoxydihydrodamsin (**3**, R_t_ = 9.53 min), costunolide (**4**, R_t_ = 17.3 min), and isoalantolactone (**5**, R_t_ = 18.3 min). The HPLC chromatograms of the crude extracts were evaluated at 210 nm (Figure S2B). In the HPLC chromatograms, baseline separation could be achieved for two compounds (**1** and **3**), while near-baseline separation was achieved with three compounds (**2**, **4**, and **5**) present in minor amounts (Figure S2C,D).

The calibration curves are based on 8–10 calibration points. The correlation coefficients of the calibration curves were at least 0.997. The mixture of the reference standards was injected four times to assess the suitability of the analytical system. The low relative standard deviation (RSD%) values of the area under the curves (AUCs) and retention times, together with the tailing factors below, confirmed that the system was suitable for the measurement of these compounds (Table 1).

### 2.2. Quantification of Sesquiterpene Lactones

**Figure 1:**
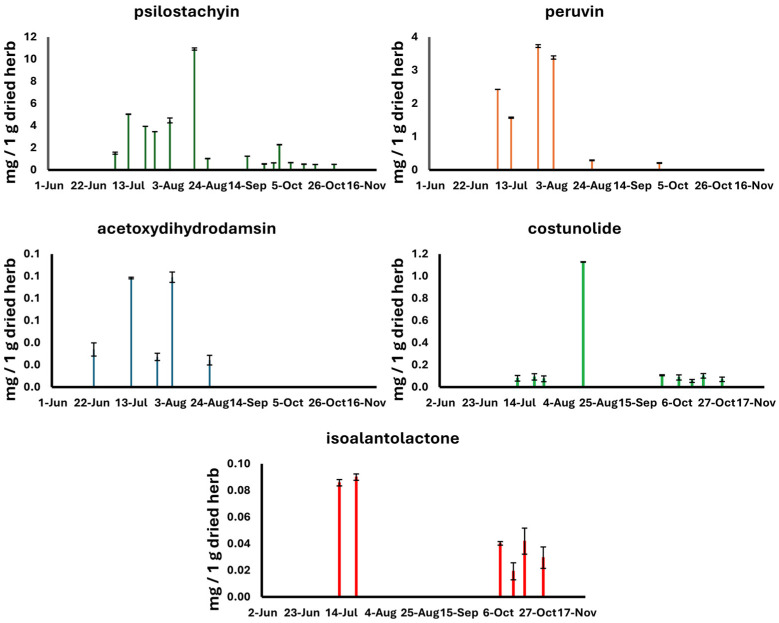
Concentration levels of sesquiterpene lactones in the aerial parts of *A. artemisiifolia* collected near Szeged from June to October.

**Figure 2:**
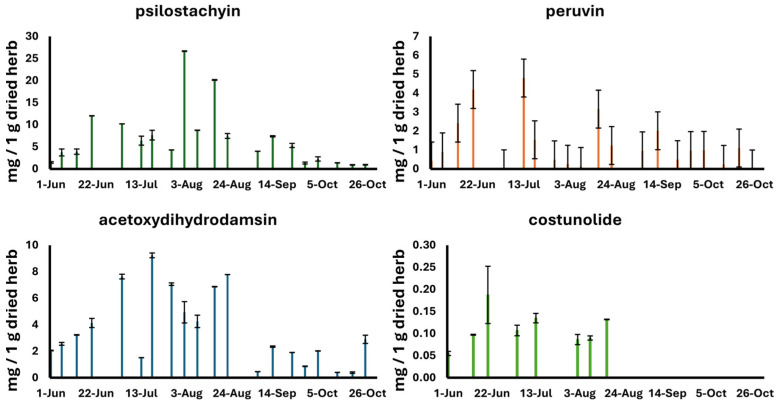
Concentration levels of sesquiterpene lactones in the aerial parts of *A. artemisiifolia* collected near Nyíri from June to October.

The amounts of the targeted marker compounds in the aerial parts of ragweed samples collected near Szeged and Nyíri are presented in Table 2 and Table 3, respectively. Figure 1 and Figure 2 graphically present the variations in the observed compounds over time. The aerial parts of the ragweed collected from June to October near Szeged contained mainly psilostachyin (**1**) and peruvin (**2**) and, in a smaller amount, acetoxydihydrodamsin (**3**), costunolide (**4**), and isoalantolactone (**5**) in the range of 0.02–0.10, 0.210–3.729, 0.499–10.917, 0.0561–1.128, and 0.019–0.090 mg/g, respectively. In the set of samples collected near Nyíri, psilostachyin (**1**) was observed in the highest amount (26.66 mg/g), together with peruvin (**2**) (4.80 mg/g), while the compounds acetoxydihydrodamsin (**3**) and costunolide (**4**) were observed with minor concentration ranges, and isoalantolactone (**5**) could not be identified. In general, the amount of sesquiterpene lactones analyzed was lower in the plant material samples collected near Szeged compared to those collected near Nyíri. The differences were significant (*p* < 0.001) for psilostachyin (**1**), peruvin (**2**), and acetoxydihydrodamsin (**3**), while the difference in the concentration levels of costunolide (**4**) was not significant.

Characteristic trends were observed in the variations in the sesquiterpene lactone concentrations throughout the analyzed time range. In general, the concentrations peaked in the range from the end of July to the middle of August. Differences in the sesquiterpene variation profiles could be identified in the sample sets with different geographical origins. The ragweed collected near Szeged contained elevated concentrations in the second part of July and in the first part of August for the main compounds (i.e., psilostachyin (**1**) and peruvin (**2**)). Interestingly, psilostachyin (**1**) and a minor compound, costunolide (**4**), had the same variation profiles. On the contrary, the ragweed samples collected near Nyíri showed elevated sesquiterpene concentration levels in an earlier stage of the vegetational period, with a significant decrease in the first part of August for the main compounds (i.e., psilostachyin (**1**), peruvin (**2**), and isoalantolactone (**5**)).

The psilostachyin (**1**) content, the major constituent of the aerial parts of ragweed, peaked in the beginning of August in the samples collected near Szeged and in the middle of August in the samples collected near Nyíri. The samples collected near Szeged showed a steady elevation until the middle of August followed by a drastic drop at the end of August. The Nyíri samples showed different profiles for this compound. A moderate elevation in the level of psilostachyin (**1**) was followed by peaks at the beginning and in second part of August, respectively, and finally, the level of this compound gradually decreased until the end of October. The highest concentration of psilostachyin (**1**) was observed in the sample collected in Nyíri, with a value of 26.66 mg/g. On the contrary, the samples collected in the same period of summer near Szeged contained much lower quantities of psilostachyin (**1**) (10.92 mg/g). Isoalantolactone (**5**) was identified as a minor compound, with a concentration level lower than the limit of quantification (LOQ) in the samples collected near Szeged; however, it was absent in the samples collected near Nyíri. On the contrary, costunolide (**4**) was present as a minor compound in a very low amount (0.06–1.13 mg/g) in both samples. The quantity of the compounds analyzed in the ragweed samples were the highest in the bloom period, from the end of June (23 June 2021) until the last weeks of September (21 September 2021), at both collection sites (Figure 1 and Figure 2).

The acetoxydihydrodamsin (**3**) content analyzed in the samples collected near Nyíri was markedly different, ranging from 0.39 to 9.23 mg/g in all the samples from June to October. The highest amount of the above-mentioned sesquiterpene in the aerial parts was detected in the samples collected in the middle of July, whilst the samples from the last weeks of October contained the lowest concentration (0.39 mg/g) (Figure 2). The content of peruvin (**2**), the third major compound, ranged from 0.01 mg/g (4 July) to 4.80 mg/g (13 July) in the samples collected near Nyíri. In comparison, the samples collected in Szeged contained the highest amounts at the end of July (3.73 mg/g) and in the first weeks of August (3.38 mg/g) (Figure 1).

The statistical analysis of the sample sets collected in Szeged and Nyíri demonstrated that the differences were significant in terms of the concentration levels of psilostachyin (**1**) (t(2) = 6.32, *p* < 0.001), peruvin (**2**) (t(2) = 3.69, *p* < 0.001), acetoxydihydrodamsin (**3**) (t(2) = 11.93, *p* < 0.001), and costunolide (**4**) (t(2) = 6.32, *p* < 0.001), respectively. No isoalantolactone (**5**) could be detected in the Nyíri samples.

**Figure 3:**
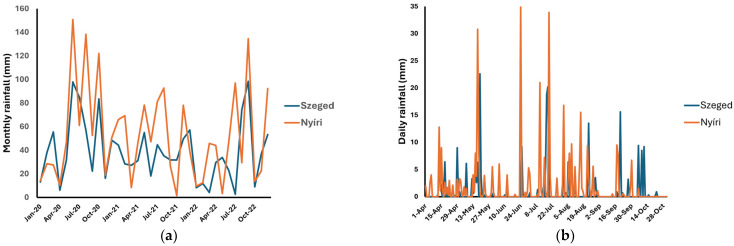
Monthly average rainfall recorded from 2020 to 2022 (**a**) and daily rainfall recorded from April to October in 2021 (**b**). Data source: HungaroMet.

**Figure 4:**
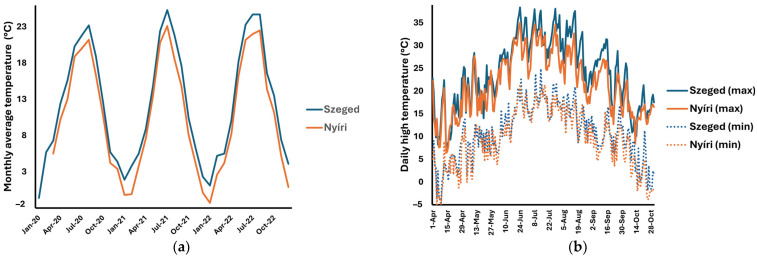
Monthly average temperatures registered from 2020 to 2022 (**a**) and the daily lowest and highest temperatures registered from May to October in 2021 (**b**). Data source: HungaroMet.

**Figure 5:**
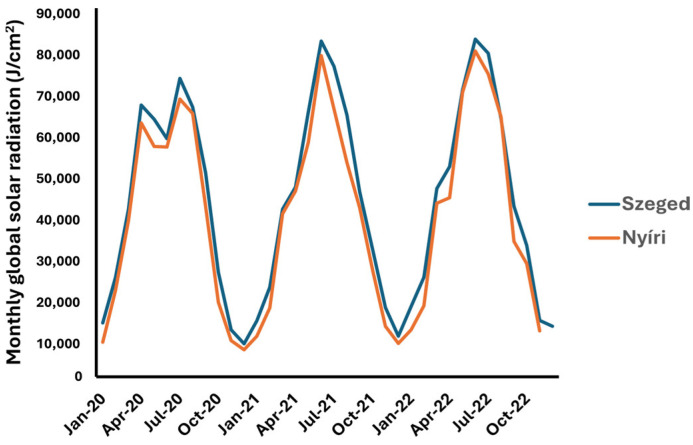
Monthly global solar radiation recorded at collection sites. Data source: HungaroMet.

The Meteorological Database of the Hungarian Meteorological Service (http://odp.met.hu, accessed on 1 February 2024) was the source of the climatic parameters, including the daily temperature (average, minimal temperature, and maximal daily temperature), global radiation, and daily precipitation. These data are presented in Figure 3, Figure 4 and Figure 5. Correlations between climatic factors and sesquiterpene lactone concentration levels were analyzed using Pearson’s correlation. The correlation data are presented in Table 4. The results indicated a weak-to-moderate positive correlation between the temperature and sesquiterpene lactone concentration levels. A weak correlation could be observed in the Nyíri samples. Interestingly, a weak negative and non-significant correlation was observed between the daily rainfall and sesquiterpene lactones in the Szeged sample set; however, this correlation must be interpreted with the fact that in the observed period, it only rained for one day in Szeged. There was no correlation with global radiation.

## 3. Discussion

*A. artemisiifolia* generally grows in dense populations, which facilitates the collection of substantial biomass in a small space. The collection from the different sites in Hungary (northeast and southeast) provided plants with different phenotypes. The samples from the northeast part of Hungary had much denser foliage covered with a dense glandular trichome layer, and the samples from the blooming period also bore a denser composite inflorescence. In contrast, the plant samples collected from the southeast part of Hungary had less-dense foliage and capitulum and higher stems. These differences in the phenotype between the two collection sites might partially explain the observed differences in the sesquiterpene lactone profiles, since in Asteraceae plants, the sesquiterpene lactone synthesis is located in the smooth endoplasmic reticula of capitate glandular trichomes (CGTs), and it is then secreted in the extracellular and subcuticular space [13]. Until now, most sesquiterpenes have been isolated from the aerial parts of *A. artemisiifolia*, demonstrating the observation that the key enzymes of sesquiterpene biosynthesis are located in the smooth endoplasmic reticulum of CGT secretory cells located on the leaf surface [14]. In accordance with these observations, the variation pattern of the sesquiterpene lactone concentration levels might correlate with the development stage of aboveground organs (i.e., foliage density and trichome coverage). The aerial parts of the Szeged samples at the beginning of the harvesting period were fully developed, with an average height of 50 cm, while the Nyíri sample set had an average height of 30 cm. The ongoing harvesting yielded samples with less-dense foliage, and they consequently had a lower trichome coverage in Szeged. In contrast, the Nyíri sample set was more developed, with an average foliage density and trichome coverage.

Geoclimatic parameters could have a further effect on the specialized metabolites of ragweed, resulting in significant differences in the sesquiterpene lactone profiles of ragweed samples collected at different geographical locations. Taglialatela-Scafati and his colleagues also reported that even collections from sites with a distance of only 100 m afforded different phytochemical profiles of *A. artemisiifolia* collected in Novara, Italy; the first collection was characterized by the presence of different sesquiterpene profiles with hydroxydihydrodamsin as the main compound, and the third collection had isabelin as the main compound. The authors hypothesized that this was due to the remarkable genetic variation in *A. artemisiifolia* [15]. Božičević and his colleagues investigated the aerial parts and pollen of ragweed from the same collection place (Novara, Italy) [16]. The authors pointed out that the *Ambrosia* pollen did not contain sesquiterpenes that were present in the leaves and flowers, as Taglialatela-Scafati and his co-workers reported from the same collection places. These findings also demonstrate the genetic variation in ragweed and how this phenomenon may alter specialized plant metabolites in plants in different seasons, even from the same collection places.

Although the content and composition of these types of specialized metabolites may differ regarding the harvesting place, vegetation period, etc., some studies have reported the isolation some of the main characteristic sesquiterpene lactones of the genus. Ragweed samples collected in the US [17], Australia [18], and Argentina [19] led to the isolation of psilostachyin (**1**). Similarly, the collection of samples of *A. artesmisiifolia* from Poland [20] and Italy [15] resulted in a similar chemical profile, demonstrating the isolation of peruvin (**2**) and acetoxydihydrodamsin (**3**) as additional characteristic *Ambrosia* sp. sesquiterpene lactones from both collection sites, demonstrating that these types of terpenoids are widely distributed in species of different origins [16].

*A. artemisiifolia* belongs to a genus with a complex phylogeny, where species variability is augmented by several factors like hybridization [21], which creates hybrid herbs; there is some evidence of introgression within *A. artemisiifolia* and *A. psilostachya*. This gradually allows genes to move from one species to the gene pool of another, when there is an opportunity for hybridization [22]. Self-pollination and self-fertility are other factors that provide these plants with strong inbreeding. This enables micro-populations to undergo rapid genetic drifts, even when smaller populations are found in regions where the species is generally abundant [23]. The genetic factors mentioned above can influence the chemical profile of the plant, including the content of specialized metabolites in the same habitats.

In addition to genetic factors, some geoclimatic elements may also have impacts on the sesquiterpene lactone content of ragweed. These specialized metabolites may play a role as potential stress alleviators to help plants adapt to environmental stressors, forcing detrimental impacts on plant health and survival. Environmental stresses in plants may lead to significantly reduced growth as well as physiological and yield responses, eventually leading to early senescence and/or cell death [24]. A wide range of environmental conditions are altered with the height of the natural growth location. Such conditions include the mean temperature, precipitation, soil, extreme temperatures, wind speed, duration of snow cover, duration of the vegetation period, and radiation intensities, such as ultraviolet (UV)-B irradiation. Among these, water stress is a major issue in managing plant development and production. Zhang and his colleagues evaluated the effects of soil moisture regimes, which corresponded to the effect of the soil water-holding capacity on *Chrysanthemum morifolium* Ramat. (Asteraceae) and its inflorescence morphology and medicinal ingredients [25]. The authors observed that a higher soil water level helped to generate more specialized metabolites (3,5-dicaffeoylquinic acid and chlorogenic acid), and flooding stress during flower–bud differentiation significantly increased the accumulation of specialized metabolites. A study conducted with a phylogenetically close species, *Artemisia annua* L., revealed that the content of the main sesquiterpenes (artemisinin, arteannuin-B, artemisinic acid, and dihydroartemisinic acid) was negatively modulated, while some minor monoterpenes and all sesquiterpenes as essential oil components were induced by water deficit treatment [26]. The authors also noted that water deficit stress decreased the glandular trichome density and size, which correlates with our observations: the ragweed samples collected near Szeged had a much lower glandular trichome density compared to the samples from Nyíri. Our results also showed similarities with the findings of others on the developmental profile of metabolites in *A. annua*; the sesquiterpene concentration reached a peak at full flowering [27]. In addition to these Asteraceae species, rainfall seems to be one of the crucial factors affecting the vegetative development of ragweed and its pollinosis as well. In Hungary, Pinke et al. reported a strong correlation between April precipitation and ragweed infestation [28], suggesting that significantly higher territories were covered by ragweed in cases where the April precipitation exceeded 39 mm. Case et al. reported a significant impact of August rainfall on ragweed pollination, indicating that precipitation extremes were negatively correlated with pollen production [29]. The average monthly rainfall from 2020 to 2022 and the daily rainfall of 2021 are presented in Figure 3, indicating a low monthly precipitation level for 2021. April totaled 31.2 mm (20 dry days) and 46.2 mm (16 dry days) of precipitation in Szeged and Nyíri, respectively. August was 50% drier in Szeged compared to the average Hungarian rainfall registered in the period of 1991–2020, while in Nyíri, the rainfall totaled 90 mm compared to the average 60 mm. Furthermore, the frequency of rain and the amount of precipitation increased from the end of July 2021 until the end of August 2021. Our analysis revealed a weak correlation between precipitation and the production of psilostachyin (**1**), acetodydihydrodamsin (**3**), and constunolide (**4**), respectively, in the Nyíri sample set. The obvious differences in water supply could be one of the determinants of a lower content of sesquiterpene lactones in the samples collected near Szeged. It could be hypothesized that the increase in precipitation could have had an effect on the elevation of the sesquiterpene lactone content in the samples collected between the end of July and the end of August.

Optimal growth conditions for *A. artemisiifolia* have been studied and reported in the literature, including temperature. The minimum temperature for normal plant growth is 22 °C [30]; however, the optimal temperature regime is 26–30 °C [31], and the plant terminates its growth above 43 °C [32]. The analysis of the meteorological data registered closest to the origin of our samples allowed us to conclude that the average monthly temperatures in spring and summer of 2021 were slightly higher than in previous years (Figure 4a), especially in Szeged. The analysis of the lowest and highest temperature for the period from May to October 2021 (Figure 4b) revealed that the highest daily temperature exceeded the optimal value for ragweed, but it remained below 35 °C. A weak-to-moderate correlation could be observed between the daily temperature and the examined sesquiterpene lactones, except isoalantolactone (**5**), which was present in very low amounts in the Szeged samples.

The monthly global solar radiation was slightly higher in Szeged than in Nyíri (Figure 5); however, the data in the literature suggest that daylight is positively correlated with pollen emission [33].

Ragweed has a wide tolerance for soil composition. It grows in nutrient-rich soils as well as in saline soils. In Hungary, it occurs more frequently on sandy and loessal soils [34]. The tolerance for salinity of ragweed was investigated by Skálová et al. in laboratory studies. Ragweed biomass was determined to decrease significantly at a higher salinity (75 mmol NaCl), while a lower salinity was optimal (25 mmol NaCl) for biomass production [30]. The ragweed samples investigated were grown on fertile soil rich in humus and nutrients. The samples near Szeged were growing in chernozem, while near Nyíri, the soil is brown forest soil.

Our findings based on the HPLC analysis suggest that ragweed has a high variability in terms of its sesquiterpene content. These types of specialized metabolites reached their peak during the full-bloom period, from mid-July to mid-August, in the samples from both collection sites (Figure 1 and Figure 2). Flowers could be observed in the first quarter of July, and the full-bloom period was from mid-July to mid-August in the samples from both collection sites, in accordance with previous reports [35]. Ripened fruits appeared from mid-October [36]. Our results are in great agreement with the findings reported by other research groups [19]. It seems that the sesquiterpene lactone content might be influenced by various geoclimatic factors; therefore, herbs from different geographical locations are different in terms of their specialized metabolite profiles. Furthermore, the observed differences might be explained as an extremely rapid and successful adaption of ragweed to new environmental conditions [37].

## 4. Materials and Methods

### 4.1. Chemicals

HPLC-grade solvents (MeCN, acetone, and MeOH) were acquired from Chem-Lab NV (Zedelgem, Belgium); water for the HPLC analyses was purified using a Millipore Direct-Q^®^ 3 UV Water Purification System (Millipore S. A. S., Molsheim, France). Trifluoroacetic acid was purchased from VWR (Debrecen, Hungary; 99%). The sesquiterpene lactones psilostachyin (**1**), peruvin (**2**), and acetoxydihydrodamsin (**3**) were isolated by our group [38]; costunolide (**4**) was obtained from Merck KGaA (Darmstadt, Germany; purity >97%), while isoalantolactone (**5**) was purchased from MedChemExpress LLC (Monmouth Junction, NJ, USA; purity 99.99%).

### 4.2. HPLC Analysis and Quantification

#### 4.2.1. Sample Preparation

The aerial parts of *A. artemisiifolia* were collected from June to October 2021 in the suburban regions of Szeged (southeast Hungary, N 46.28, E 20.14, height above mean sea level: 70 m) and Nyíri (northeast Hungary, N 48.50, E 21.44, height above mean sea level: 240 m). Botanical identification was performed for the main anatomical characteristics of the species *Ambrosia artemisiifolia* var. *elatior*, which is solely present in Hungary [39]. In the early stages, the leaves are always pinnate, and the plant is green or dark green in color. In this stage, *A. aretmisiifolia* might be misidentified with *Artemisia vulgaris;* however, in the case of young *A. vulgaris*, the blade of lower leaf is less divided (i.e., more lobed), and the margin is serrate. At the developed stage, the ragweed has erect stems that are glabrous to rough hairy. The leaves are opposite below, alternate above, and tri-pinnatifid. The uppermost leaves are occasionally different, having unlobed blades compared to the lower leaves. Ragweed flowers, as a monoecious plant, contain either male or female flowers in their flower heads [36,39].

The harvested plant material was dried at room temperature in a shaded, well-ventilated location for one week. Voucher specimens of the collected herbal samples (Szeged samples: 2021/VI-20 to 2021/VI-39; Nyíri samples: 2021/VI-40 to 2021/VI-60) were deposited in the herbarium of the Institute of Pharmacognosy, University of Szeged. The aerial parts of the dried raw plant material (100 g) were ground using a blender Retsch Grindomix GM 200 (Retsch GmbH, Haan, Germany). A total of 10 g of the powdered plant material was extracted with 100 mL of acetone two times separately for ten minutes in an ultrasonic bath at room temperature. The extract was filtered using filter paper (Whatman 4 filter papers, diameter 125 mm) and centrifuged for 5 min at 5000 rpm using a Rotanta 460 Hattich (Kirchlengem, Germany) centrifuge. The supernatant was quantitatively transferred into a rounded-bottom flask, and the solvent was evaporated in vacuo (Büchi Labortechnik AG, Flawil, Switzerland). The dry residue was redissolved in 20 mL of MeOH (HPLC-grade), transferred into a volumetric flask, and diluted to 25 mL. Before HPLC analysis, each sample was filtered through a syringe filter with a polytetrafluoroethylne (PTFE) membrane (d = 13 mm, porosity: 0.45 µm) (Nantong FilterBio Membrane Co., Ltd., Nantong City, China) and immediately injected. The extractions were carried out in triplicates.

#### 4.2.2. HPLC Analysis

The HPLC analyses were performed using a Shimadzu LC20 Liquid Chromatograph (Shimadzu Corp., Kyoto, Japan) equipped with SPD-M20A Diode Array Detector (DAD), degasser unit, automatic injector, auto sampler, and a column oven and operated by a CBM-20A Communication Bus Module (Shimadzu Corp., Kyoto, Japan).

Separations were performed on a reverse-phase Kinetex^®^ C_18_ column (5 µm, 100Å, 150 × 4.6 mm, Phenomenex, Torrance, CA, USA) equipped with a guard column (4.6 × 10.0 mm) with the same packing material. The mobile phase A was water with 0.1% (*v*/*v*) TFA, and mobile phase B was acetonitrile with 0.1% (*v*/*v*) TFA. The chromatographic elution of the samples was carried out with a gradient solvent system as follows: 20% MeCN (0–1 min), 20-70% MeCN (1–19 min), 70–100% MeCN (19–21 min), kept at 100% MeCN for 2 min, 100 to 20% (23–24 min), and kept at 20% for 6 min at a flow rate of 1.4 mL/min; the temperature of the column was set at 30 °C; the injection volume was 10 µL.

The UV-DAD detector was set to record between 205 and 800 nm, and the chromatograms were recorded at 210 nm to detect the targeted compounds.

The calibration curves were generated using a methanolic dilution series of the reference standards containing acetoxydihydrodamsin (**1**), peruvin (**2**), psilostachyin (**3**), costunolide (**4**), and isoalantolactone (**5**), respectively. In general, dilution series of the reference compounds were prepared starting from 1 mg/mL, resulting in three concentration levels (1, 0.1, and 0.01 mg/mL), respectively. By injection of an appropriate volume of the solutions, the linear calibration plots were obtained at eight to ten different concentration levels, covering the linear range presented in Table 1. All the calibration levels were measured four times. Linear regression was used to establish the calibration curve. The results were calculated using the AUC.

### 4.3. Meteorological Data

Data on the rainfall, average temperature, and global radiation were downloaded from the Meterological Database of the Hungarian Meteorological Service (http://odp.met.hu, accessed on 1 February 2024). Data registered at meteorological station Szeged-külterület (No. 58116) [40,41] were used to describe the conditions for the sample set collected near Szeged, while for the Nyíri sample set, the average temperature and rainfall registered by the nearest meteorological station Hidasnémeti (No. 61104) [42,43] and the global solar radiation data registered by the station nearest Sátoraljaújhely (No. 61709) [44,45] were analyzed. Daily and monthly data from 2020 to 2022 were used to observe trends in the rainfall, average temperatures, and global solar radiation.

### 4.4. Statistical Analysis

The normal distribution of the data was checked using the Shapiro–Wilk test. The *T*-test or one-way ANOVA with Bonferroni’s post hoc test were used to determine whether there were any statistically significant differences between the investigated samples. Pearson’s correlation coefficient was computed to determine the relationship between the meteorological data and sesquiterpene lactone concentration levels. The differences or correlations were considered significant when *p* < 0.05 (*), *p* < 0.01 (**), and *p* < 0.001 (***). Statistical analyses were carried out using R (version 4.0.3, The R Foundation for Statistical Computing, Vienna, Austria, http://www.r-project.org, accessed on 1 July 2024).

## 5. Conclusions

Sesquiterpene lactones are specialized plant metabolites with promising pharmacological activities. These metabolites are characteristic marker compounds for *Ambrosia artemisiifolia.* Numerous sesquiterpene lactones have been isolated from ragweed’s aerial parts; however, there is no information on their bioproduction and quantification throughout the life cycle of the plant. Here, we report for the first time a quantitative analysis of sesquiterpene lactone production in ragweed. Significant differences in the amount and bioproduction rhythm of sesquiterpene lactones were found between two sets of samples. The concentration levels of the analyzed sesquiterpene lactones were significantly lower in the samples collected near Szeged compared to the Nyíri samples. The highest concentration levels were observed at either the end of July or in August. A weak-to-moderate correlation could be established between the production of the analyzed sesquiterpene lactones and climatic factors (i.e., precipitation and temperature). Geographical location and geoclimatic factors might exert significant influence on the production of sesquiterpene lactones in ragweed.

## Figures and Tables

**Table 1:** Characteristics of the calibration curves and limit of detection and quantification values, and results of the system suitability test. (LOD: limit of detection, LOQ: limit of quantification, RSD: relative standard deviation, RT: retention time).

Standard	LOD (µg/inj)	LOQ (µg/inj)	Calibration Points	Range Covered (µg/inj)	Regressions Equations	R^2^	RSD% of AUC	Tailing Factor	RSD% of RT
psilostachyin (**1**)	0.04155	0.12590	9	0.02–20.00	y = 621593.7970x − 150728.6813	0.9970	1.00	0.876–1.224	0.16
peruvin (**2**)	0.06877	0.20841	9	0.05–4	y = 4929518558x − 46339884	0.9999	0.78	0.947–1.216	0.11
acetoxydihydrodamsin (**3**)	0.02688	0.08121	10	0.02–5	y = 1178552.7162x + 52571.8051	0.9992	0.54	1.081-1.110	0.13
costunolide (**4**)	0.17653	0.53496	8	0.02–2.5	y = 417313.4111x − 32627130	0.9999	0.78	1.108–1.789	0.12
isoalantolactone (**5**)	0.07902	0.23947	8	0.02–2.5	y = 851443.9139x + 6909.0185	0.9999	0.29	1.108–1.239	0.11

**Table 2:** Contents of marker compounds in aerial parts of *A. artemisiifolia* collected near Szeged, Hungary.

Date of Harvest	Psilostachyin	Peruvin	Acetoxydihydrodamsin	Costunolide	Isoalantolactone
2 June 2021	ND	ND	ND	ND	ND
8 June 2021	ND	ND	ND	ND	ND
15 June 2021	ND	ND	ND	ND	ND
23 June 2021	ND	ND	<LOQ	ND	ND
6 July 2021	1.52 ± 0.10	2.43 ± 0.00	ND	ND	ND
13 July 2021	5.03 ± 0.02	1.57 ± 0.02	0.10 ± 0.00	<LOD	<LOQ
22 July 2021	3.94 ± 0.00	ND	ND	<LOD	<LOQ
27 July 2021	3.46 ± 0.01	3.73 ± 0.04	0.03 ± 0.00	<LOD	ND
4 August 2021	4.47 ± 0.22	3.38 ± 0.05	0.10 ± 0.01	ND	ND
17 August 2021	10.92 ± 0.10	ND	ND	1.13 ± 0.00	ND
24 August 2021	1.03 ± 0.01	0.03 ± 0.01	<LOQ	ND	ND
14 September 2021	1.25 ± 0.00	ND	ND	ND	ND
23 September 2021	0.54 ± 0.03	ND	ND	ND	ND
28 September 2021	0.64 ± 0.01	0.21 ± 0.01	ND	<LOD	ND
31 September 2021	2.26 ± 0.03	ND	ND	ND	ND
7 October 2021	0.66 ± 0.02	ND	ND	<LOD	<LOD
14 October 2021	0.53 ± 0.02	ND	ND	<LOD	<LOD
20 October 2021	0.50 ± 0.00	ND	ND	<LOD	<LOD
30 October 2021	0.51 ± 0.01	ND	ND	<LOD	<LOD

The values are presented as mean ± SD (n = 3) of marker compound amounts in mg/1 g of dried aerial parts of the herb. (LOQ: limit of quantification, LOD: limit of detection, ND: not detected).

**Table 3:** Contents of marker compounds in aerial parts of *A. artemisiifolia* collected near Nyíri, Hungary.

Date of Harvest	Psilostachyin	Peruvin	Acetoxydihydrodamsin	Costunolide	Isoalantolactone
1 June 2021	1.46 ± 0.19	0.42 ± 0.02	2.06 ± 0.02	<LOD	ND
6 June 2021	3.73 ± 0.79	0.90 ± 0.01	2.60 ± 0.10	ND	ND
13 June 2021	3.91 ± 0.60	2.42 ± 0.03	3.23 ± 0.02	<LOD	ND
20 June 2021	12.01 ± 0.03	4.19 ± 0.04	4.13 ± 0.34	<LOD	ND
4 July 2021	10.23 ± 0.00	<LOD	7.63 ± 0.18	<LOD	ND
13 July 2021	6.39 ± 1.02	4.80 ± 0.00	1.51 ± 0.00	<LOD	ND
18 July 2021	7.64 ± 1.11	1.54 ± 0.03	9.23 ± 0.17	ND	ND
27 July 2021	4.30 ± 0.03	0.48 ± 0.01	7.06 ± 0.10	ND	ND
2 August 2021	26.66 ± 0.08	0.25 ± 0.01	4.94 ± 0.81	<LOD	ND
8 August 2021	8.76 ± 0.04	<LOQ	4.25 ± 0.47	<LOD	ND
16 August 2021	20.13 ± 0.09	3.16 ± 0.08	6.87 ± 0.02	<LOD	ND
22 August 2021	7.47 ± 0.56	1.24 ± 0.00	7.78 ± 0.01	ND	ND
5 September 2021	4.00 ± 0.01	0.96 ± 0.01	0.46 ± 0.00	ND	ND
12 September 2021	7.39 ± 0.14	2.02 ± 0.03	2.35 ± 0.05	ND	ND
21 September 2021	5.32 ± 0.48	0.49 ± 0.00	1.90 ± 0.00	ND	ND
27 September 2021	1.32 ± 0.22	0.97 ± 0.00	0.86 ± 0.02	ND	ND
3 October 2021	2.26 ± 0.48	0.98 ± 0.04	2.02 ± 0.01	ND	ND
12 October 2021	1.37 ± 0.03	0.24 ± 0.03	0.40 ± 0.00	ND	ND
19 October 2021	0.91 ± 0.06	1.11 ± 0.16	0.39 ± 0.06	ND	ND
25 October 2021	0.94 ± 0.07	ND	2.90 ± 0.31	ND	ND

Values are presented as mean ± SD (n = 3) of marker compounds in mg/1 g of dried aerial parts of the herb. (LOQ: limit of quantification. LOD: limit of detection. ND: not detected).

**Table 4:** Correlation data between climatic factors and concentration levels of sesquiterpene lactones.

Climatic Factor	Collection Site	Psilostachyin (1)	Peruvin (2)	Acetoxydihydrodamsin (3)	Costunolide (4)	Isoalantolactone (5)
temperature (average)	SZEGED	0.34	0.59 (**)	0.55 (*)	0.00	0.01
	NYÍRI	0.46 (*)	0.46 (*)	0.58 (**)	0.61 (**)	ND
temperature (minimal)	SZEGED	0.46 (*)	0.52 (*)	0.50 (*)	0.18	−0.09
	NYÍRI	0.59 (**)	0.43	0.61 (**)	0.54 (*)	ND
temperature (maximal)	SZEGED	0.24	0.55 (*)	0.52 (*)	0.09	−0.26
	NYÍRI	0.43	0.45 (*)	0.55 (*)	0.62 (**)	ND
global radiation (daily)	SZEGED	0.04	0.33	0.30	−0.18	0.02
	NYÍRI	0.12	0.39	0.35	0.46	ND
precipitation (daily)	SZEGED	–0.12	–0.12	–0.11	−0.01	0.20
	NYÍRI	0.53 (*)	0.02	0.21	0.37	ND

Values are Pearson’s correlation coefficients for Szeged (df = 17) and Nyíri (df = 18) sample sets. The correlation is significant if *p* < 0.05 (*) or *p* < 0.01 (**). (ND: not detected).

## Data Availability

The original contributions presented in this study are included in the article; further inquiries can be directed to the corresponding author.

## Supplementary Materials

The following supporting information can be downloaded at: https://www.mdpi.com/article/10.3390/plants13152053/s1, Figure S1: Sesquiterpene lactones isolated from *A. artemisiifolia* used as target compounds for HPLC analysis; Figure S2: HPLC chromatogram of the reference compounds (A) and the crude extract (B), with focus on compounds **1**–**3** (C) and compounds **4**–**5** (D) at 210 nm.
