# Supplementary figures and images for: Targeted Screening and Quantification of Characteristic Sesquiterpene Lactones in Ambrosia artemisiifolia L. at Different Growth Stages
Source: Plants (Basel). 2024 Jul 25;13(15):2053. doi: 10.3390/plants13152053 (PMC11314284; doi:10.3390/plants13152053)

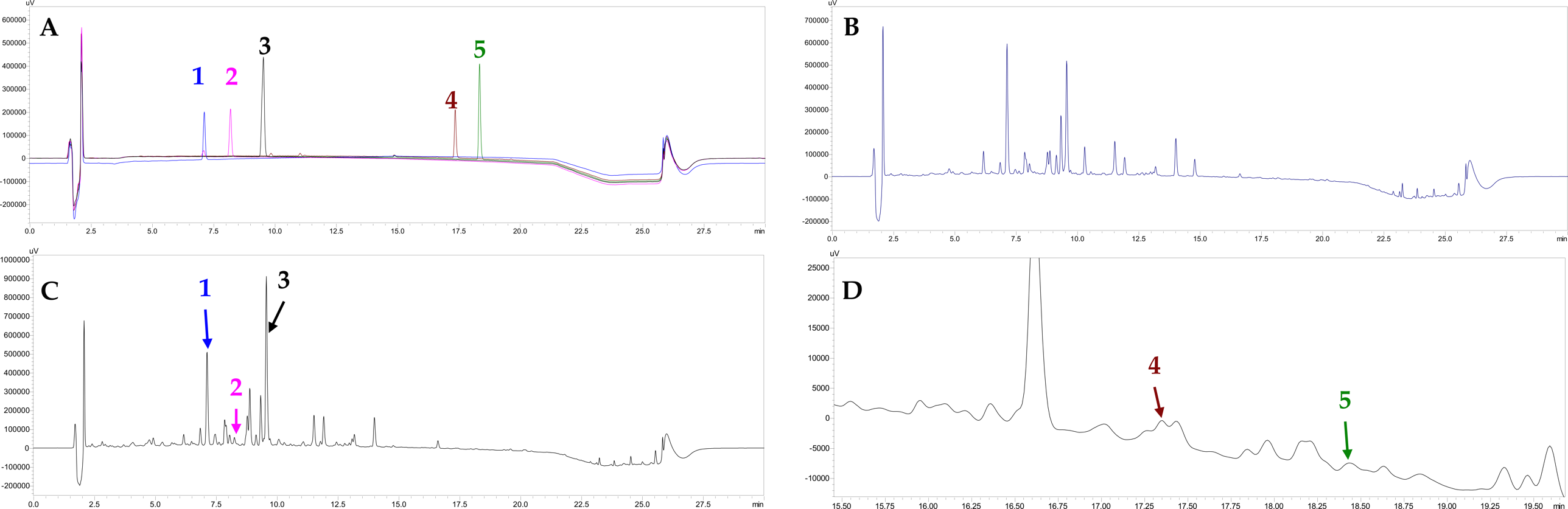

Supplement: Supplementary file 1 [file plants-13-02053-s001.zip › FigS2.tiff]
